# The chemokine receptor CCR5: multi-faceted hook for HIV-1

**DOI:** 10.1186/s12977-024-00634-1

**Published:** 2024-01-23

**Authors:** Natacha Faivre, Christel Verollet, Fabrice Dumas

**Affiliations:** 1 Institut de Pharmacologie et de Biologie Structurale (IPBS), Université de Toulouse, CNRS, Université Toulouse III – Paul Sabatier (UPS), Toulouse, France; 2 International Research Laboratory (IRP) CNRS “IM-TB/HIV”, Toulouse, France; 3 International Research Laboratory (IRP) CNRS “IM-TB/HIV”, Buenos Aires, Argentina

**Keywords:** HIV-1, CCR5, Chemokine receptor, Conformation, Lipid membrane

## Abstract

Chemokines are cytokines whose primary role is cellular activation and stimulation of leukocyte migration. They perform their various functions by interacting with G protein-coupled cell surface receptors (GPCRs) and are involved in the regulation of many biological processes such as apoptosis, proliferation, angiogenesis, hematopoiesis or organogenesis. They contribute to the maintenance of the homeostasis of lymphocytes and coordinate the function of the immune system. However, chemokines and their receptors are sometimes hijacked by some pathogens to infect the host organism. For a given chemokine receptor, there is a wide structural, organizational and conformational diversity. In this review, we describe the evidence for structural variety reported for the chemokine receptor CCR5, how this variability can be exploited by HIV-1 to infect its target cells and what therapeutic solutions are currently being developed to overcome this problem.

## Introduction

Chemokines belong to the cytokine family. Their chemoattractant properties are at the origin of their name (chemoattractant cytokines). They are a family of small, soluble 8–14 KDa proteins that exert important functions of intercellular communication. All chemokines exert their functions by binding to G-protein coupled receptors (GPCR) also called chemokine receptors. Chemokines, regulate many biological processes such as proliferation, apoptosis, angiogenesis, hematopoiesis or lymphoid organ development, but their main role is to activate and control leukocyte migration. Following interaction with their specific chemokine ligands, chemokine receptors trigger signaling that initiates a chemotaxis process, which traffics the cell to a desired location within the organism. These molecules are involved in the maintenance of lymphocyte homeostasis under physiological conditions and the deregulation of their expression is associated to many diseases [1]. Although chemokine receptors play a pivotal role in the host's antimicrobial defense mechanisms, they can serve as primary targets for various pathogens. These pathogens may either encode chemokine mimics or, as observed in the context of HIV-1, use chemokine receptors to directly infect their target cells.

Some pathogens can induce the production of molecules that bind to receptors with a higher affinity than their natural ligands [2–5], others will modify the expression level of chemokines [6, 7] or of chemokine receptors [8]. In all cases, this is an effective way to either evade immune selection or, on the opposite, to lead to a deleterious burst of the immune system (cytokine storm) [9]. This strategy is used by a wide variety of pathogens ranging from ticks [10, 11], parasites [12, 13] to bacteria [6, 7, 14] and viruses [15–30]. Among viruses, one can cite as examples Epstein-Barr viruses that encode a GPCR (BILF1) capable of forming a dimer with CXCR4 chemokine receptor thereby inhibiting its signaling but also human cytomegalovirus (CMV) that secretes a soluble chemokine receptor (pUL21.5) which binds selectively to the chemokine RANTES with very high affinity, blocking the interaction of RANTES with its cellular receptors (CCR5). In both cases, viruses interfere with cellular signaling processes, reducing the effectiveness of the immune response [17]. Such phenomenon has also been described for poxviruses such as Respiratory syncytial virus [18], mousepox [19, 20], smallpox [21, 22], monkeypox [23] (of which several hundred cases have been diagnosed in humans in Europe during the spring 2022), herpes viruses [24–27] and HIV [28]. This is not an exhaustive list and for more details see [29, 30].

In some cases, direct attachment of pathogens to a chemokine receptor represents the first step of infection, the receptor acting as an anchor that allows the pathogen to dock to the plasma membrane of its target cell before entering into the cell or releasing its contents into the cytoplasm. This has for instance been described for pathogenic bacteria Staphylococcus aureus (whose toxins interacts with CCR5 chemokine receptor) [31], for Human Respiratory Syncytial Virus (that binds to CX3CR1 chemokine receptor) [32–34] and HIV (that uses CCR5 and/or CXCR4 chemokine receptors to attach and infect immune cells) [35–38]. In the case of HIV-1, the CCR5 chemokine receptor not only acts as an anchor for the virus to attach to the cell surface, but also plays an active role in the infection process by initiating signaling cascades that mimics the signaling of chemokines and promotes infection [28, 39–41]. To be thorough, and although it is outside the scope of this review, it is interesting to note that the HIV can also bind to other receptors such as glycan-binding proteins, so-called lectins, which mediate potent viral transmission to CD4^+^ T cells known as *trans*-infection [42]. One can cite DC-SIGN/CD209 [43], the C-type lectin DC immunoreceptor (DCIR) [44], the mannose receptor (MR) or Siglec-1/CD169 [45].

A very large number of studies have been conducted on HIV-1 and have revealed the ability of this virus to exploit the wide structural variability (i.e. different conformational states) of chemokine receptors to bypass the immune system and circumvent some therapeutic approaches. It is this last aspect that we aim to discuss in this review: how the structural diversity of chemokine receptors is exploited by HIV-1 to infect its target cells, whether this diversity comes from one person to another, from one cell type to another or within the same cell. We will here focus mainly on the C-C chemokine receptor type 5, also known as CCR5 and to a lesser extent on C-X-C chemokine receptor type 4 (CXCR4).

## HIV-1 infection requires interaction with chemokine receptors

**Fig. 1:**
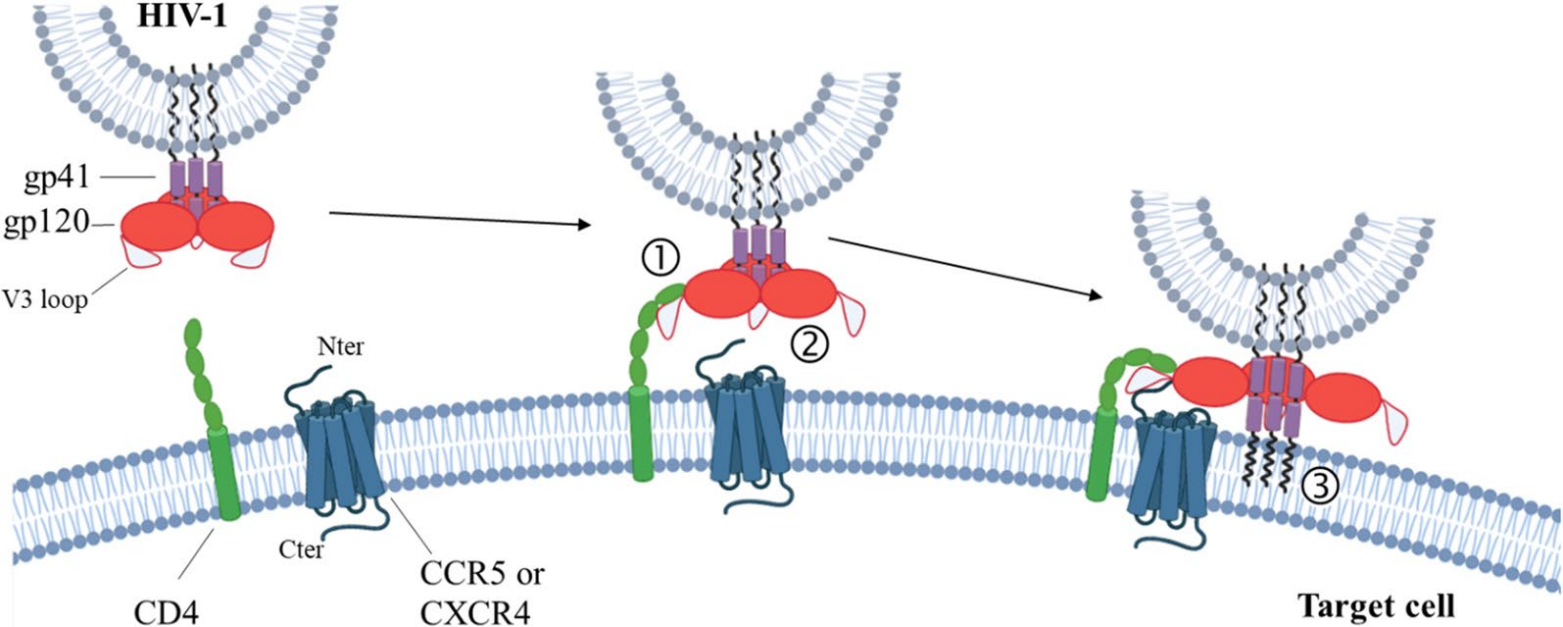
Schematic view of the early steps of HIV infection. The HIV envelope (Env), consisting of gp120 and gp41 subunits, first binds to the CD4 receptor located at the plasma membrane of the infected cell (1). This binding induces a conformational change in gp120 that allows the interaction of its V3 loop with a co-receptor (2). The co-receptor can be either CCR5 or CXCR4, defining the tropism of the virus (see main text). This second binding induces a new conformational change in gp120 which exposes the fusion peptide of gp41 that anchors its N-terminal domain into the plasma membrane of the target cell (3). This initiates a process of fusion that will lead to the release of the viral content into the cytoplasm of the infected cell (for more details see [38, 54])

The early steps of HIV-1 infection first involve the binding of the HIV protein envelope gp120 to the CD4 receptor (Fig. 1, step 1). This process then requires the interaction of gp120 with a chemokine receptor than can be either CCR5 or CXCR4 (Fig. 1, step 2). This second interaction defines the tropism of the virus infecting the cell; viruses that bind to CCR5 are called “R5”, those that bind to CXCR4 are called “X4” and those that are able to bind to both are known as “R5X4”. R5 viruses are preferentially transmitted [46] and are predominant during the chronic phase of infection [47, 48]. Approximately 50% of patients with advanced HIV develop X4 virus in addition to R5 virus. This usually occurs after several years and is associated with a decline in the CD4 + T cell count and a rapid progression to a pathologic state [49–53].

The combination of the structural diversity of both HIV-1 and CCR5 offers a broad range of possible interactions between these molecules, providing as many opportunities for infection. On the one hand, the V3 loop of the viral protein gp120, which is involved in the binding to CCR5 (Fig. 1, step 2) [55, 56], is one of the most variable sites of HIV-1, with differences of up to 50% between isolates [57, 58]. This explains why the number of CCR5 molecules recognized by different HIV-1 gp120 proteins varies differently between various cell types [59]. On the other hand, GPCRs have long been depicted as switches that can exist in two distinct states: inactive and active. Over the past two decades, this vision has considerably evolved and it is now well established that the situation is far more complex than originally thought. Numerous crystallographic and cryo-electron microscopy structures have revealed that GPCRs can adopt many different three-dimensional structures. Ligands or G-proteins selectively bind to a subset of these conformations, causing a population shift and establishing a new equilibrium [60–64]. In this way, Urvas and Kellenberger recently published a comparative analysis of all chemokine—chemokine receptor structures uncovering that while N termini receptors structures exhibit conserved interaction patterns with chemokines, the second extracellular loop exhibits several subfamily-specific features [65]. Moreover, these structures are only the tip of the iceberg since they represent only static snapshots of the most stable states of the proteins when there is a thermal equilibrium which leads to oscillations between multiple transient conformations [66].

## Structural variability of chemokines receptors involved in HIV-1 infection

CCR5 and CXCR4 receptors belong to the GPCR class A family. These cell-surface receptors can detect extracellular molecules and induce an intracellular response. Binding of signal molecules to the extracellular surface of the receptor activates intracellular G proteins which in turn act on various effectors mediating intracellular signaling effects. These receptors are made up of 7 transmembrane helices connected by three extracellular and three intracellular loops and their overall 3D structure can be modulated by many factors. Importantly, the 3D structure of CCR5 and CXCR4 associated with different antagonists has been elucidated a decade ago [67, 68].

### Post-translational modifications

**Fig. 2:**
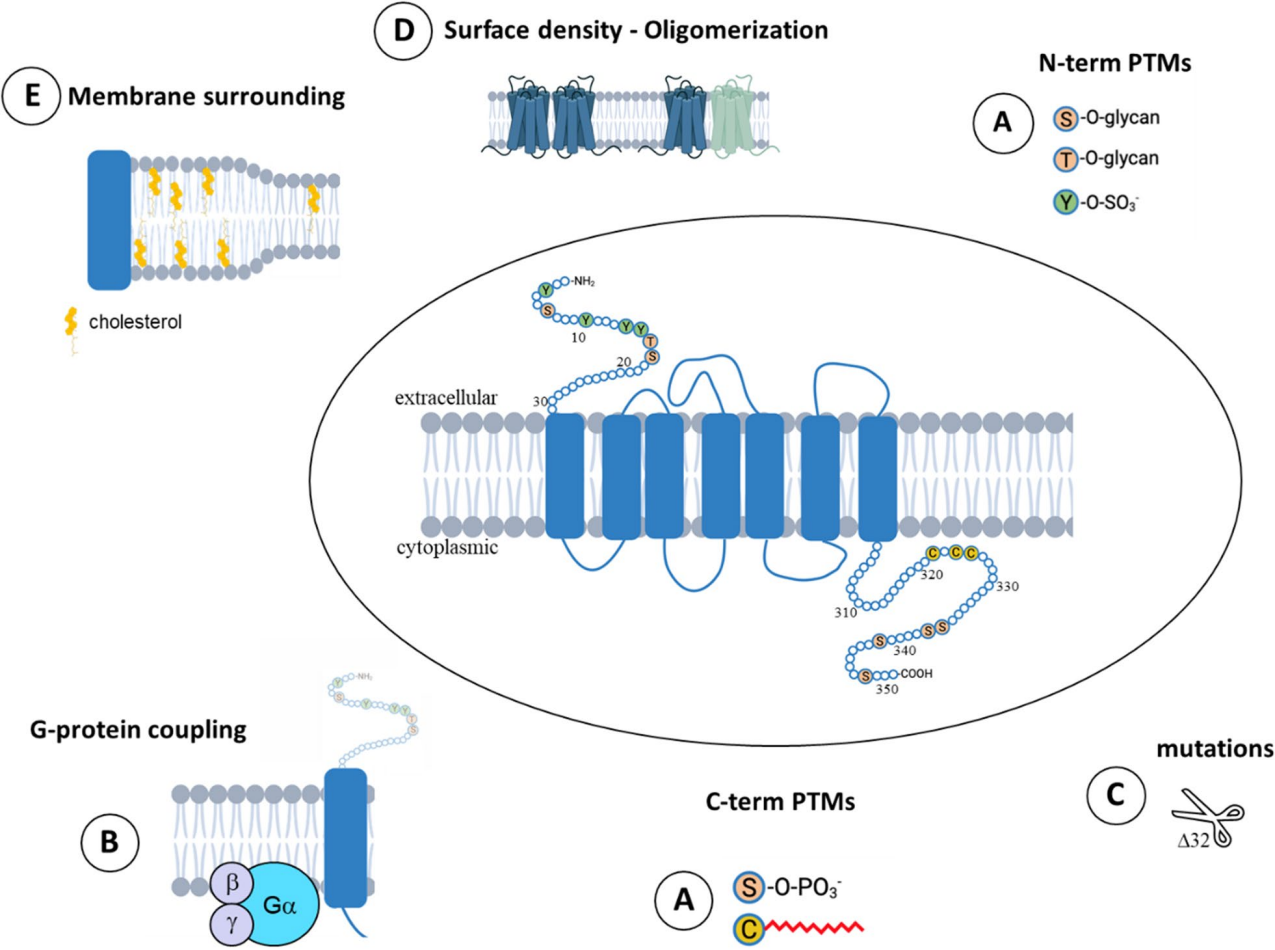
Schematic representation of the factors that can influence the 3D-structure of CCR5 coreceptor. **A** Post-translational modifications (PMTs): sites of O-Glycosylation (Ser6, Thr16, Ser17) [70, 71], sulfation (Tyr3, 10, 14, 15) [73, 75–78], palmitoylation (Cys321, 323 and 324) [79–91] and phosphorylation (Ser336, 337, 342 and 349) [235, 236] are represented by colored circles. Factors such as G-protein coupling (**B**) [28, 60, 97–103], mutations (**C**) [126–129, 177, 178], surface density [117–123] and oligomerization [136–149] (**D**) and membrane surrounding [152–173] (**E**) are also exemplified

A first level of structural differences relates to the modulation of post-translational modifications of receptors. Hundreds of post-translational modifications of proteins have been reported to date [69]. This diversity leads to a structural variety of proteins that will influence their ability to interact with their ligands but also offer many possibilities for viruses to adapt to their attachment. Many post-translational modifications have been described for CCR5 including, o-glycosilation, phosphorylation, sulfation and palmitoylation (Fig. 2A).

*O-glycosylation* and Phosphorylation—O-glycosylation of CCR5 does not appear to affect the ability of HIV-1 to infect cells [70] and there is little data on the impact of CCR5 phosphorylation on HIV-1 infection. Phosphorylation of this receptor appears to be primarily involved in internalization and recycling and thus its abundance at the surface of the cell (for review, see [71]). However, one cannot exclude that it also has an impact on the overall structure of the proteins and contributes to their structural diversity at the surface of the cell.

*Sulfation*—By contrast, sulfation of tyrosine residues 3, 10, 14 and 15 of the N-terminal moiety of CCR5 has been described to play an important role in the functionality of this receptor [72]. In 1999, Farzan and colleagues have shown that sulfation of the amino-terminal part of CCR5 contributes to the binding of its ligands MIP-1 α and MIP-1 β but also to its binding to gp120/CD4 complexes and thus to the ability of HIV-1 to infect cells [73]. More recently, using ELISA approaches and performing binding experiments on cells expressing CCR5 under conditions that modulate their sulfation level, Scurci and colleagues have shown that CCR5 sulfation is heterogeneous thereby affecting the binding properties of natural chemokines and some monoclonal antibodies (mAb 3A9 only binds to the sulfated peptide while mAb HEK/1/85a shows a clear preference for the unsulfated peptide) [74]. These authors also suggest that the difference in binding ability between natural ligands (MIP-1 α, MIP-1 β, RANTES/CCL5) and the analogue 5P12-RANTES (an HIV entry inhibitor that binds to CCR5) are—at least in part—due to the heterogeneity of sulfation on the cell surface (other factors, such as the molecular fit of ligands with the transmembrane domain of the receptor, can also influence their global affinity). This proposal is in agreement with other recent NMR works that have identified sulfation combinations that are important for ligand binding [75–77]. To a lesser extent this effect is also observed with CXCR4, which exists in several different forms of sulfation [78].

*Palmitoylation*—CCR5 can also be palmitoylated on three cysteine residues: C321, C323, and C324, in the C-terminal tail of CCR5 [79, 80]. The presence of palmitate groups will affect CCR5 at three levels: on the structure of the receptor itself, on its cell distribution and on its membrane localization [81, 82]. From a strictly structural perspective, the insertion of palmitate into the cytoplasmic leaflet of the plasma membrane profoundly modifies the conformation of the cytosolic tail housing the palmitoylated cysteine [83] and affect the overall GPCR structure [84–86]. In addition, such modification has been shown to promote coupling to G-receptors [87] and phosphorylation of CCR5 [88] leading to a potential increase of structural diversity of the receptors. Another important effect of palmitoylation lies in its impact on the membrane distribution of receptors within the cell. Palmitoylation of membrane proteins is often required for their correct folding, which protects them from the degradation pathway associated with the endoplasmic reticulum. In other words, palmitoylation orchestrates the subcellular traffic of GPCRs to the plasma membrane. For this reason, HIV-1 cannot infect cells in which CCR5 is not palmitoylated, because it is not addressed to the plasma membrane [79, 89]. The presence of a saturated lipid (i.e. palmitate) that inserts into the membrane is known to promote protein relocation into liquid-ordered raft domains [86, 90, 91]. In this regard, palmitoylation of CCR5 is essential for the incorporation of CCR5 into the plasma membrane rafts [85] that offers a new lipid environment to the proteins inducing some conformational changes that will be discussed in the following section of this manuscript.

### Membrane organization of chemokine receptors

As mentioned before, chemokine receptors present a structural diversity linked to their intrinsic chemical composition. Beyond this strictly “structural” aspect, the receptor surrounding plays an essential role in the modulation of its three-dimensional structure. Among these environmental factors, the coupling with G proteins or other transmembrane proteins but also the interactions with their lipid environment are key elements.

*Coupling to G proteins*—As indicated by their name, GPCRs can be coupled (or not) with different types of heterotrimeric G proteins (Fig. 2B). Human genome encodes 18 different Gα proteins, 5 Gβ proteins, and 12 Gγ proteins [92]. CCR5 has been shown to interact with several of them: Gαi, Gαq, Gβ and Gγ [28, 93]. This combination of possible interactions not only contributes to the structural diversity of CCR5, offering larger possibilities of HIV to bind to the cells, but it also favors infection by triggering signal transduction upon HIV binding.

Conformational changes: many authors have studied if interactions with G proteins could modify CCR5 conformation and affect their ability to interact with HIV-1. Certain small molecule inhibitors of CCR5 used as HIV-1 inhibitors, such as vicriviroc (VVC), maraviroc (MVC) or TAK779, act as allosteric modulators that stabilize a conformation of CCR5 that the virus does not effectively recognize, thus preventing its entry [94–96]. Several authors have investigated whether the binding of these inhibitors was affected by the coupling of CCR5 with G proteins. By performing binding assays, they showed that the association of the intracellular domains of CCR5 with G proteins alters their interactions with the inhibitors, suggesting that the association of CCR5 with the signaling machinery induces substantial conformational changes that affect the binding of these inhibitors and their ability to block HIV-1 entry [97–100]. Similar conclusion were also obtained by other studies that used monoclonal antibodies able to identify different CCR5 conformations [101]. These proposals were corroborated by cryo-electron microscopy structures which showed notable structural changes depending on whether CCR5 was coupled to a Gαi1 protein or not [102]. This is also in agreement with former results obtained on another GPCR receptor, the β2-adrenergic receptor, whose coupling with the G protein is loose and does not stabilize the receptor in a single conformation but allows for the existence of distinct intermediates [60, 103]. Taken together, these results show that whether or not CCR5 is coupled to G proteins is a source of conformational diversity.

Signal transduction: binding of HIV-1 gp120 to CCR5 has been shown to trigger activation of Gαi or Gαq proteins. This results in the modulation of many important cellular functions, including cell migration, adhesion, or survival [104–112]. Additionally, it is noteworthy that the interaction between the viral envelope and CCR5 can also trigger a response via β-arrestins. Initially considered for their role in receptor desensitization, β-arrestins are now recognized as versatile scaffolds interacting with various signaling proteins, including MAPKs, PI3K, and protein kinase B [113]. The activated signaling pathways will ultimately impact the cell susceptibility to HIV infection. For instance, it has been demonstrated that exposure to viral gp120 can initiate viral replication in cultures of resting CD4 T cells from infected individuals [114]. These findings suggest that virus-hijacked signaling process may subsequently facilitate pathogenesis. More details of these processes can be found in reviews by Wu and Yoder [28], Nickolof-Bybel et al. [40] and Juno and Fowke [115].

The intricate interplay between the G proteins, the CCR5 receptor and its binding to the HIV envelope protein underscores the complexity of understanding the exact role of each in HIV infection. The ability of the virus to infect its target cells depends not only on its ability to bind to the receptor, but also on its ability to trigger a cellular response that promotes its spread. Therefore, elucidating not only the ability of the virus to bind to CCR5, but also the signaling pathways it induces in conjunction with these structural changes could open the door to the discovery of innovative antiretroviral agents.

*Receptors surface density*—HIV-1 infection involves lateral organization of the receptors as it requires the binding of trimers of the virus envelope glycoprotein to host cell receptors leading to membrane fusion. Numerous studies have shown that effective infection can only occur if several of these trimers are simultaneously involved in the process [116]. As a consequence, the density of receptors present at the surface of the cells will have a direct impact on the capacity of the viruses to infect them [117–121] (Fig. 2D). For example, by quantifying CCR5 expression in acutely HIV-infected subjects over a 2 year period, it was shown by Yang et al. that high levels of CCR5 on CD4 central memory cells are associated with rapid disease progression [122]. Conversely, in elite controllers (individuals who spontaneously control HIV-1 replication in the absence of antiretroviral therapy), the analysis of the surface phenotype and transcriptional profile of CD4 + T cells has revealed low CCR5 expression, leading to the conclusion that low CCR5 expression protects the cells from HIV-1 infection [123]. Finally, it has been shown that some mutations induce a decrease for CCR5 addressed to the plasma membrane, conferring to individuals an almost total resistance to HIV-1 infection. This is the case for truncated CCR5 due to the delta 24 mutation [124, 125] and the delta 32 mutation [126–129] (Fig. 2C) that are known to impact the localization of CCR5. This will be developed in the next chapter. Various studies have then investigated the minimum number of receptors required for infection. Infectivity assays performed on HeLa cells in which individual clones express either large or small amounts of CD4 and distinct amounts of CCR5 have shown that the minimum amount of CCR5 allowing infection is dependent on the quantity of CD4 in the membrane and vary from 1.10^3^ to 1.10^4^ CCR5 per cell [119]. Furthermore, approximately four to six CCR5s assemble around the virus to form a complex needed for infection [130]. Since the efficiency of infection depends on both CD4 and CCR5 concentrations rather than on precise amounts of each, the authors have proposed that this efficiency also depends on the diffusion of these proteins within the membrane and on their ability to encounter each other to form virus-CD4-CCR5 ternary complexes [37]. This was later on evidenced by Baker et al. who measured the dynamics of CD4 and CCR5 by FRAP and revealed the existence of membrane domains that concentrate CD4 and CCR5 in a 1:5 ratio [131]. Finally yet importantly, it appears that the levels and expression profile of CCR5 can influence the tropism of virus strains for different cell types [132–134] and correlates with the appearance of viral reservoirs [135].

*Oligomerization of receptors*—Many GPCRs are able to interact with each other to form higher-order structures (homomers and/or heteromers) (Fig. 2D) that can, upon allosteric modulation, exhibit distinct biochemical properties from monomers [136–139]. Both CCR5 and CXCR4 have been shown to form many different homo and heteromers whose exhaustive lists can be found in [140] (Table 1) and [141] (Table 1). Many studies have used Bioluminescence or Fluorescence Energy Transfer (BRET and FRET, respectively) to detect oligomers formation. These methods are based on the fact that light energy can only be transferred from a donor to an acceptor if these molecules are less than 10 nm apart. By using these experimental approaches, it has first been shown that CCR5 constitutively forms homodimers at the surface of T-lymphocytes [142]. Thereafter, similar fluorescence measurements combined with the use of monoclonal antibodies that recognize different epitopes of CCR5 allowed to establish that CCR5 oligomers are structurally different from monomers and that HIV-1 preferentially recognize the monomeric form of the receptor [143]. Similar results have been obtained by dynamic approaches consisting in tracking and classifying the motion of different receptor subpopulations [144, 145]. Further studies finally provided evidence of three distinct CCR5 dimeric organizations [146]. On the same principle, the formation of CCR5-CCR2b heteromers has been evidenced and binding experiments revealed that the interaction between heterodimer units is of allosteric nature and modifies the binding properties of chemokines [147, 148]. Interestingly, oligomerization of CCR5/CD4/CXCR4 receptors has also been reported in the literature. This association has been shown to prevent X4 HIV-1 virus from binding to target cells [149]. Overall, these data support the view that oligomerization of chemokine receptors affects their structure and may influence the susceptibility of cells to infection. The development of super-resolution fluorescence methods, such as Stochastic Optical Reconstruction Microscopy (STORM) or Photo-Activation Localization Microscopy (PALM) has permitted direct visualization of GPCRs such as mu-opioid receptor [150] and dopamine receptor [151] at the single-molecule level, unveiling that their oligomers are transitory structures with rapid association and dissociation kinetics. This opens the gate to future experiment carried out on CCR5 and CXCR4 to decipher their mechanism of oligomerization and its role in HIV-1 infection.

*Lipid surrounding*—Finally, it has long been known that the lipid membrane has an impact on the structuring of membrane proteins [152, 153] (Fig. 2E), this is particularly true for GPCRs [154–156], including CCR5 and CXCR4 [157–160]. For example, it has recently been reported that ceramide or related sphingolipids might invert the topology of CCR5, preventing macrophages from migrating toward CCL5 [161] and similar results were obtained with CXCR4 and the CXCL12 chemokine [160]. Concurrently, Calmet et al. have reconstituted CCR5 receptors into model membranes of controlled lipid composition and demonstrated that cholesterol decreases the binding affinity of maraviroc to CCR5 [157]. Molecular dynamics simulation further suggested that cholesterol restricts the structural dynamics of the receptor [157]. It is interesting to notice that sphingolipids and cholesterol are key components involved in the formation of lipid rafts [162, 163]. Actually, a lot of work has been done to understand the contribution of lipid rafts in the organization of HIV-1 receptors and in the infection process [164]. Indeed, extensive studies conducted in the late 1990s have shown that the CD4 receptor is clearly associated with rafts [165–167]. Subsequently, other studies based on raft extraction and protein analysis have shown that CCR5 is mainly associated with lipid rafts [168] while CXCR4 is only partially localized into them [168–170]. Moreover, the use of conformational antibodies has revealed that rafts stabilize the active conformation of CCR5 towards its ligands [171]. Lastly, Yang and colleagues have used model systems mimicking HIV-1 envelopes and T-cell membranes to demonstrate that the hydrophobic mismatch at boundaries between liquid-ordered and liquid-disordered regions (i.e. lipid raft and non-raft regions) generates a line tension that can facilitate membrane fusion with the virus envelope [172, 173]. Taken together, these data suggest that lipid rafts may act as platforms that facilitate virus entry both by fostering local concentrations of receptors and/or co-receptors and by modulating their conformation.

### Genetic mutation of chemokine receptors is double-edged sword for HIV-1

In this review, we have discussed the beneficial effects that heterogeneity in chemokine receptor structure could have on HIV-1 infection. Nevertheless, this variability can, in some rare cases, be detrimental for viruses. Indeed, it has been found in the 90’s that about 1% of the Caucasian human population carries a homozygous 32 base pair deletion in the gene encoding for the CCR5 receptor, resulting in a truncated gene that produces non-functional receptors named CCR5 delta-32 [127, 132, 174]. This mutation, discovered in a group of homozygous patients known as "exposed uninfected", confers resistance to infection. It has been shown that the protein CCR5 delta-32 remains mostly localized in the endoplasmic reticulum where it exerts a trans-dominant negative effect on the wild type CCR5 protein, preventing its transport to the cell surface [175]. Furthermore, it forms dimers with the CXCR4 proteins in the ER, which also limits their distribution to the plasma membrane [176]. As a consequence, both CCR5 and CXCR4 are absent from the plasma membrane conferring to the cells a resistance to R5, X4 and R5X4 viruses [126–129]. This observation has been exploited to initially cure two patients who became famous and known as " Berlin patient" [177] and " London patient " [178]. Both of them have received allogeneic hematopoietic stem cell transplantation from donors with a homozygous CCR5 delta-32 mutation to cure a leukemia [179]. More recently, three other patients have been cured using the same strategy, one at Weill Cornell hospital of New York in the frame of IMPAACT P1107 clinical trial (NCT02140944) [180], a second one at the City of Hope cancer institute in Los Angeles as revealed by Jana Dickter during the AIDS 2022 press conference and a third one in Düsseldorf in 2023 [181]. At this stage, it should be stressed that we cannot exclude the possibility that additional factors, such as the transplant procedure itself, may have played a role in these results.

Although large-scale therapy based on this strategy is not feasible, these results demonstrate that CCR5 is a suitable target for HIV-1 gene therapy. It should be noted that such a treatment could potentially influence other medical conditions. For instance, it has been suggested that the CCR5-delta32 mutation might contribute to prolonged kidney transplant survival [182]. Conversely, it has also been proposed that this mutation could increase susceptibility to neuroinvasive West Nile virus infections [183].

## Targeting CCR5 to fight the virus

In the last decades, three main strategies targeting CCR5 have mainly been developed to block HIV infection: the use of CCR5 ligands, the use of antibodies and, more recently, editing the genome in order to eradicate CCR5 from the cell surface or modify its structure. It is important to highlight that the first two strategies can take advantage of the structural diversity of CCR5 by specifically targeting the subset of receptors implicated in HIV-1 infection. This targeted approach may help to minimize the adverse effects of drugs. In the light of the structural variabilities of CCR5 discussed above, we will now discuss the potential of each of these strategies.

*Development of CCR5 ligands*—This strategy aims to develop molecules that will bind to CCR5 and prevent the interaction between CCR5 and the gp120 HIV protein thereby aborting HIV entry mediated by fusion and infection. Up today, many molecules have been produced such as Tak-779 [96], cenicriviroc [184], CMPD-167 [185], Aplaviroc [186], vicriviroc [94] or maraviroc [95] (for a detailed review see [187, 188]). Despite the large number of molecules developed and clinical trials carried out, only maraviroc has been approved for use in HIV treatment in 2007 [189]. It remains the only CCR5 inhibitor in clinical use to date. It is an inverse CCR5 agonist that impairs HIV envelope binding to CCR5 through a noncompetitive and allosteric modulation [67, 95, 98, 190].

However, nine CCR5 mutations have been identified as inducing a decrease of the affinity for maraviroc (up to tenfold) [191]. It should be noted that, with one exception, all these mutations also modify gp120 binding and are therefore less detrimental in terms of protection against viral infection. The use of maraviroc can cause a tropism switch to X4 strains although this is a very rare event in the absence of pre-existing X4 strains. This drug cannot be used in the presence of X4 strains, as a result, it requires a viral tropism test before use. Part of the problem of its use also arises from the possible emergence of virus variants that recognize the maraviroc-CCR5 complex, as well as free CCR5 [192–194].

*Development of anti-CCR5 antibodies*—Various attempts have been made to develop antibodies that prevent HIV infection [195–197]. These antibodies are designed not only to bind to CCR5 and prevent its interaction with the viral protein gp120, but also to trigger the internalization of the co-receptor [198]. Only two antibodies reached clinical trials: CCR5mAb004 [188], that have been withdrawn in 2006, and Leronlimab [196] (formerly PRO 140), for which many clinical trials have been completed (NCT02175680, NCT00110591, NCT02483078, NCT00613379, NCT00642707) or are still running (NCT03902522, NCT02859961, NCT02355184, NCT02990858, NCT05271370). This antibody has not yet been granted approval by the Food and Drug Administration, but has been designated fast-track status in 2019 [199]. Leronimab is a humanized monoclonal IgG4 antibody that targets the extracellular domain 2 of CCR5, and impairs its interaction with HIV-1 gp120 preventing virus entry. Subcutaneous administration of this antibody to HIV-1-infected individuals significantly reduced their viral load between successive injections [200]. When administered intravenously to HIV-1 infected adult subjects, it has also showed potent and long-lived antiviral activity [201]. The only downside is that Leronimab does not recognize all CCR5 proteins since receptor occupancy was around 85% [201] meaning that 15% of CCR5 receptors potentially escape to the antibody and might participate to the emergence of resistant HIV-1 strains. Their use also raises pharmacokinetic issues. Because they are proteins, they cannot be absorbed from the gastrointestinal tract and must be administered parenterally. A final issue arises from the fact that the use of anti-CCR5 antibodies can lead to a shift in tropism towards CXCR4 [202].

*CCR5 gene editing*—Recent advances in genome editing offers unprecedented possibilities for gene therapy, as it is now possible to replace one or more bases in any gene of interest, at any location [203, 204]. The CRISPR/Cas9 system or the use of zinc finger nucleases (ZFN) are methods of choice for such gene editing [205, 206]. These tools can be very useful to fight against HIV infection. The experimental strategy consists in extracting hematopoietic stem cells from patients, suppressing CCR5 expression in these cells thanks to an appropriate treatment (i.e. CRISPR/Cas9 or ZFN) before reinjecting them. The treated cells, newly resistant to infection, should then be able to proliferate and substitute cells sensitive to HIV-1. Several attempts have been performed in the frame of clinical trials such as NCT03164135 (CCR5 depletion thanks to CRIPR/CAS9), NCT03666871 and NCT02388594 (CCR5 depletion thanks to ZFN). These experiments have been shown to produce in vitro HIV-1 resistance which constitutes an encouraging proof of concept for HIV cure [207–211]. Despite these successes for CCR5 ablation, low genome-editing frequencies [212] and high off-target activity[213] have been observed. Therefore, genome-editing techniques still need to be improved before they can be used on a mass scale. Furthermore, since CCR5 deletion alone has no impact on infection by X4-tropic viruses, some authors have performed a double knockout (KO) CCR5 + CXCR4 [214]. Unfortunately, a poor engraftment of the R5X4-CRISPRCas9 KO CD4 + T cells in mice bone marrow has been obtained.

Another promising approach consists in taking advantage of the delta-32 mutation described earlier. This time, the strategy consists in modifying CCR5 gene (and not suppress it) in order to express the delta-32 truncated CCR5 protein. We have seen that this mutation suppresses the expression of CCR5 and CXCR4 at the cell surface, thus protecting them from R5- and X4-tropic viruses. This also present the advantage to target one gene instead of two and the risk of adverse side-effects seems limited insofar as this mutation has been observed naturally on thousands of people who do not seem to present any deleterious effects. Using this method, Ye et al. [215] and Qi et al. [216] succeeded to render cells resistant to HIV-1 infection whatever the tropism of the virus. Again unfortunately, the successful rate was low (20% on Jurkat cells and 11% in primary CD4 + cells) [216] so the protocols still need to be improved. Unlike the other approaches discussed in this chapter, CCR5 delta 32 editing overcomes the structural variability of CCR5 described in this manuscript. As such, this strategy appears to be a very promising approach for eradicating HIV by targeting CCR5.

## Conclusion

The more varied the receptor structure, the more difficult it is to block them all, and the more opportunities viruses will have to bind to them to infect cells [123, 217]. The factors that can modulate the conformation and the organization of receptors involved in HIV-1 infection are numerous: post-translational modifications, expression level, coupling to G proteins, oligomerization or mutation of CCR5 and lipid environment (Fig. 2). These factors can be interdependent which further complicates their possible structural combinations (for instance, palmitoylation promotes the localization of CCR5 into the lipid rafts domains, which will then promote their interaction with the G proteins that are also concentrated there). All these parameters can vary within the same cell [98, 218–223], from one cell type to another [99, 224–226] and, by extent, from one patient to another. The combination of these factors leads to an infinite number of intermediate conformations which will present different affinities for their natural substrates but also for HIV-1 receptors [67, 99, 100, 221, 227–230], themselves presenting a very large structural variety [59, 231–233]. This tremendous diversity explains the difficulties encountered over the past 30 years in the fight against AIDS. Considerable advances have been made and HIV infection is now well controlled by antiretroviral therapy (ART) and patients can live almost normally as long as they have access to these treatments and adhere strictly to the medication instructions. Nevertheless, these treatments do not block viral entry and they are not without risk since they can induce numerous side effects such as central nervous system disorders, bone defects, cardiovascular, hepatic or renal risks [234]. In addition, the treatments options remain limited for some patients with multiclass resistance and must be taken for life as they do not target the integrated proviral genome and fail to eradicate the virus in so called viral reservoirs. From this point of view, recent advances in genome editing offer encouraging prospects not only for blocking viral entry by modifying CCR5, but also for excising HIV sequences integrated into the genome [204]. One can hope that the combination of all these new therapeutic approaches will bring us closer to the development of an effective antiviral treatment against HIV that will be affordable for the broadest possible populations.

## Data Availability

Not applicable.
